# TRSWA-BP Neural Network for Dynamic Wind Power Forecasting Based on Entropy Evaluation

**DOI:** 10.3390/e20040283

**Published:** 2018-04-13

**Authors:** Shuangxin Wang, Xin Zhao, Meng Li, Hong Wang

**Affiliations:** 1 School of Mechanical, Electronic and Control Engineering, Beijing Jiaotong University, Beijing 100044, China; 09116318@bjtu.edu.cn (X.Z.); 16116351@bjtu.edu.cn (M.L.); 2 Department of Physics, Tangshan Normal University, Tangshan 063000, China; 3 School of Electrical and Electronic Engineering, The University of Manchester, Manchester M13 9PL, UK; mikewanguk@yahoo.co.uk

**Keywords:** wind power forecasting, TRSWA-BP, empirical mode decomposition (EMD), phase space reconstruction (PSR), normalized Renyi’s quadratic entropy (NRQE)

## Abstract

The performance evaluation of wind power forecasting under commercially operating circumstances is critical to a wide range of decision-making situations, yet difficult because of its stochastic nature. This paper firstly introduces a novel TRSWA-BP neural network, of which learning process is based on an efficiency tabu, real-coded, small-world optimization algorithm (TRSWA). In order to deal with the strong volatility and stochastic behavior of the wind power sequence, three forecasting models of the TRSWA-BP are presented, which are combined with EMD (empirical mode decomposition), PSR (phase space reconstruction), and EMD-based PSR. The error sequences of the above methods are then proved to have non-Gaussian properties, and a novel criterion of normalized Renyi’s quadratic entropy (NRQE) is proposed, which can evaluate their dynamic predicted accuracy. Finally, illustrative predictions of the next 1, 4, 6, and 24 h time-scales are examined by historical wind power data, under different evaluations. From the results, we can observe that not only do the proposed models effectively revise the error due to the fluctuation and multi-fractal property of wind power, but also that the NRQE can reserve its feasible assessment upon the stochastic predicted error.

## 1. Introduction

It is indicated that wind power possesses multifractal properties [1]. As empirical mode decomposition (EMD) can effectively deal with the problems caused by intermittent and non-stationary data [2], it has been applied to the forecasting of wind power in recent years. On the other hand, the artificial neural network combined with EMD is firstly introduced to be a reliable predictor of wind speed [3]. In order to solve the nonlinear fluctuations of the forecasting of wind power over a desired time interval, the models of EMD combined with either chaos theory or wavelet neural network have been successively brought forward [4,5]. Furthermore, a real-time prediction for wind power, based on EMD and entropy, is presented [6]. Indeed, studies have shown that the methods added to EMD have superior prediction accuracy, compared with the original methods.

Meanwhile, phase space reconstruction (PSR) has been utilized in many areas [7,8,9] since Takens proposed delay reconstruction [10]. Wolf calculated the maximum Lyapunov index of time series and first identified the existence of chaotic behavior of wind power sequences, which lay a theoretical foundation for PSR applicable to wind power sequences [11]. After this, studies of wind power forecasting based on chaotic characteristic and PSR have attracted greater attention [12]. For example, the maximum Lyapunov index of wind power sequence, on the basis of PSR, is calculated to resolve the problem of wind power short-term forecasting [13]. The chaotic characteristics of wind power parameters, from the angle of PSR optimal computation, are qualitatively analyzed, and the super short-term power prediction method is given [14]. In this context, PSR is considered a useful method to reconstruct the phase space of a dynamic system from observable variables [15,16].

However, wind power time series have a robust volatility, which can be regarded as the superposition of multiple, and aperiodic, components of disparate frequency [17]. The parametric sequence of the forecasting can be decomposed into a set of mode components which are mutually non-coupled by EMD. The component consists of chaotic variables, which affected by factors such as temperature, wind speed, air density, and humidity, etc. Therefore, the characteristic attributes of each component cannot be fully restored until PSR is completed.

Moreover, traditional error criteria based on least squares only considers second-order statistics signals, ignoring the real existence of non-Gaussian processes in the fluctuation of wind power forecasting. In this context, entropy is an effective method for analysing non-Gaussian information. For instance, by minimizing the (*h*, *φ*)-entropy of the performance index, a minimum tracking error entropy control algorithm is obtained, in order to characterize the randomness of the closed-loop system [18]. Shannon entropy is introduced to study the position and momentum of the infinite circular well [19]. The Fisher information and Shannon entropy were calculated for three position-dependent mass oscillators [20]. An entropy-based evaluation was effectively applied to medical decision support systems [21]. Consequently, the entropy expression of the forecasting error should be investigated in order to effectively evaluate the influence of non-Gaussian disturbances on the forecasting of wind power.

In view of the aforementioned analysis, this paper is organized as follows. In Section 2, a novel TRSWA-BP is constructed, where the weight of BP is trained by an efficient tabu, real-coded, small-world algorithm (TRSWA) [22]. Inspired by the chaotic behaviour of wind power sequences, the TRSWA-BP based on EMD, PSR, and EMD-based PSR are subsequently presented, in order to conquer the strong volatility and fluctuation. In Section 3, a criterion of normalized Renyi’s quadratic entropy (NRQE) is proposed, which is further designed for measuring the stochastic error in uncertain wind power forecasting, and its superiority and applicability is illustrated in detail. Section 4 examines several experimental predictions, consisting of finding solutions to different time-scales upon the data from an actual wind farm. The results demonstrate the acceptable accuracy and training times of the models, as well as the efficiency of the NRQE as a dynamic evaluation in the uncertain forecasting of wind power. Finally, our conclusions, and some possible paths for future research, are given in Section 5.

## 2. Modeling for TRSWA-BP Combined with EMD and PSR 

### 2.1. Construction of the TRSWA-BP

#### 2.1.1. Optimized Weight Iteration Calculation Based on the TRSWA

Considering the optimization problem of min*f*(*x*) (*x* ∈ *X*), the neighborhood is defined as *l*, and the Logistic chaotic map is used to generate an initial feasible solution to *x* ∈ *X* at first. Then, according to the local short-distance connection search probability, based on a kind of *n*-dimension spherical surface, the short-distance connection or non-neighborhood random long-distance connection is generated inward the small-world network neighborhood *l*, and a movement *s* ∈ *X* that can improve the current solution *x* is generated. Furthermore, in order to avoid falling into local optimum and recycle, a tabu list is constructed, which is used to store the *T_s_* movements that have just been made (*T_s_* is the length of the tabu list). Meanwhile, it is forbidden to use the movements in the tabu list during the next loop, to avoid going back to the chosen solutions. Searching is repeated until the optimal solution is found, or the stopping criterion is attained.

Suppose that a feasible solution *x* is a (*n* + 1)-dimensional vector, *x =* [*x*_1_, *x*_2_, …, *x_n_*_+1_], and the movements of the current solution *s* = [*s*_1_, *s*_2_, …, *s_n_*_+1_] are generated by Equation (1), where the phase angle *α_i_* is produced by Equation (2). The radius *r* of the nodes in the neighborhood is generated by Equation (3), and the radius *r_non_* of the nodes in the non-neighborhood is generated by Equation (4). *R* is half of the interval range, and *rand* is a random number in the interval of [0, 1].

(1) $$\left\{ \begin{array}{l} s_{1}=x_{1}+r\cos(\alpha_{1})\\ s_{2}=x_{2}+r\sin(\alpha_{1})\cos(\alpha_{2})\\ s_{3}=x_{3}+r\sin(\alpha_{1})\sin(\alpha_{2})\cos(\alpha_{3})\\ \ldots \\ s_{n}=x_{n}+r\sin(\alpha_{1})\cdot \cdot \cdot \sin(\alpha_{n-1})\cos(\alpha_{n})\\ s_{n+1}=x_{n+1}+r\sin(\alpha_{1})\cdot \cdot \cdot \sin(\alpha_{n-1})\sin(\alpha_{n}) \end{array}\right.$$

(2) $$\alpha_{i}=2\pi \times rand\;(i=1,\ldots ,n)$$

(3) r=l∗rand

(4) $$r_{non}=l+(R-l)\times rand$$

The steps of the TRSWA are described as follows.

1. Initialization is required at this stage, where the input data, including population size *Dim*, the maximum iteration *K*_max_, the temporary local network size *n_i_*, the search probability of local short-range connection *P_s_*, the size of node neighborhood radius *R_s_* and the maximum stored number of tabu list *T_s_*, are defined. Furthermore, set the number of the current iterations as *k* = 1.
2. Generate *M* (*M* > *Dim*) real-coded nodes by the Logistic chaotic map randomly, and calculate the fitness value of the objective function for each node. Find *Dim* optimal nodes among them as the initial population of node set for the TRSWA.
3. Store the searched nodes in the tabu list.
4. For each node of each generation in the node set of *Dim* population, there are *n_i_* searches of short-distance and random long-distance. Generate a random number *T_m_*. If *T_m_* < *P_s_*, perform a local short-distance search, otherwise carry out a random long-distance search. Results are compared with the saved nodes in tabu list. If the results are in the list, search again. Calculate the updated objective function and find out the optimal node set.
5. Generate a new node set, and calculate its objective function. The new node set is compared with the optimal one that has been generated in step (4), and then finds out the optimal set of the nodes. Record its location and the value of optimal fitness.
6. Check the convergence criteria. If it is satisfied, end calculation. Otherwise, let *k* = *k* + 1 and return to step 3.

#### 2.1.2. Modeling Process of the TRSWA-BP

As the TRSWA exhibits a good convergence and is a fast calculation algorithm [22], we apply it to calculate the optimized weights, in order to construct a novel BP neural network (denoted as TRSWA-BP). The BP neural network here is introduced to be a prototype model because it is similar to the multi-layered structure of a small-world BP neural network (SWBP), which has a high quality in the predictions [23]. The specific steps are as follows.

1. Determine the number of input and output nodes and the number of hidden layers and nodes in each hidden layer for the TRSWA-BP. Fix the set of training samples, and suppose *k* = 1.
2. Set parameters for the BP, such as learning rate *η* and inertia coefficient *α.*
3. Build an objective function through the training set, and the optimal weights of the BP are trained by the TRSWA.
4. Set *k* = *k* + 1. Remove the earliest set from training samples, and add a newly acquired set into it. Repeat steps 3 and 4 until the termination condition is satisfied, and a TRSWA-BP with the best weights is established.

### 2.2. TRSWA-BP Combined with EMD

EMD usually uses one kind of data sequence in wind power predictions [24,25]. We will concern five input sequences, such as data of wind speed, wind direction, wind power, temperature, and NWP wind speed, to be decomposed by EMD. The decomposed random components are used as the inputs into TRSWA-BP, to predict wind power *P*. The steps of the TRSWA-BP and EMD are described as follows.

1. Select data sequences, of which the length is *N*, including wind speed *v*(*t_i_*), wind direction *d*(*t_i_*), wind power *p*(*t_i_*), temperature *tep*(*t_i_*), and NWP wind speed denoted by *v_NWP_*(*t_i_*), *i* = 1, 2, …, *N*. Set *k* = 1.
2. *v*(*t*), *d*(*t*), *p*(*t*), *tep*(*t*), and *v_NWP_*(*t*) are respectively decomposed by EMD first, whose principle is in the order of frequency from high to low. The intrinsic modal function (IMF) of *n_v_*, *n_d_*, *n_p_*, *n_t_*, and *n_NWP_* layers, as well as a residual component *r*(·), are attained. They are denoted as *IMF*(*v*)_1_, …, *IMF*(*v*)*_nv_*, *r*(*v*), *IMF*(*d*)_1_, …, *IMF*(*d*)*_nd_*, *r*(*d*), *IMF*(*p*)_1_, …, *IMF*(*p*)*_np_*, *r*(*p*), *IMF*(*t*)_1_, …, *IMF*(*t*)*_nt_*, *r_t_*(*t*), and *IMF*(*NWP*)_1_, …, *IMF*(*NWP*)*_nNWP_*, *r*(*NWP*).
3. As the number of *n_v_*, *n_d_*, *n_p_*, *n_t_*, and *n_NWP_* may be unequal due to the decomposition, the minimum number *n*_min_ of them is taken as the number of the unified IMF layers of the five sequences.
4. If *n_v_* > *n*_min_ in the data sequence of wind speed *v*(*t*), add all the *IMF*(*v*) behind the *n*_min_th layer together with *IMF*(*v*)*_nmin_*, to form a new *IMF*(*v*)*’_nmin_*, which means that *IMF*(*v*)*’_nmin_* = *IMF*(*v*)*_nmin_* + *IMF*(*v*)_(*nmin+*1)_ + … + *IMF*(*v*)*_nv_*, (*n_v_* = 1, 2, 3, ..., *n*_min_, …, *n_v_*). The other four sequences are treated the same way, except one (or those) when n_*_ = *n*_min_. Finally, the new unified sequences are obtained according to the decomposed layers. They are *IMF*(*v*)_1_, *IMF*(*d*)_1_, *IMF*(*p*)_1_, *IMF*(*t*)_1_, *IMF*(*NWP*)_1_; *IMF*(*v*)_2_, *IMF*(*d*)_2_, *IMF*(*p*)_2_, *IMF*(*t*)_2_, *IMF*(*NWP*)_2_; …, *IMF*(*v*)’*_n_*_min_, *IMF*(*d*)’*_n_*_min_, *IMF*(*p*)’*_n_*_min_, *IMF*(*t*)’*_n_*_min_, *IMF*(*NWP*)’*_n_*_min_; and *r*(*v*), *r*(*d*), *r*(*p*), *r_t_*(*t*), *r*(*NWP*).
5. Use the first *N* − 1 data of each layer’s new unified sequence, to train several forecasting models of TRSWA-BP, respectively. The best weights are obtained by the TRSWA (See Section 2.1.2), which are employed to predict the forecasted wind power *P* of each decomposed layer. They are denoted by *P*_1_, *P*_2_, *P*_3_, …, *P_n_*_min_, and *P_r_*.
6. To compose the predictions of each layer, we obtain the fitting wind power at the *k* moment, which is given by *P_k_* = *P*_1_ + *P*_2_ + … + *P_n_*_min_ + *P_r_*.
7. Set *k* = *k* + 1. A set of newly predicted wind power *P* is used as a known value to the training set, while the earliest set of data sequences is removed. Check whether it reaches the termination condition. If not, return to step (2), otherwise stop calculation.

### 2.3. TRSWA-BP Combined with PSR

First of all, the single-variable time series {*x*(*t_i_*), *i* = 1, 2, …, *N*}, of which the sampling interval is *τ*, is conducted to the *m*-dimension vector by time delay:

(5) $$X\left(t_{i}\right)=\left(x\left(t_{i}\right),x\left(t_{i}+\tau \right),\ldots ,x\left(t_{i}+\left(m-1\right)\tau \right)\right)$$

where *i* = 1, 2, …, *M*, *m* is the embedding dimension, *τ* is the time delay, *X*(*t_i_*) is the phase point of *m*-dimension phase space, *M* is the number of phase point, and *M* = *N* − (*m* − 1)*τ*. 

Equation (5) describes the evolution trajectory of the system in the phase space. The *m* and *τ* play decisive roles on PSR, and their relationship is germane to the time window *τ_w_* of reconstructed phase space, which satisfies *τ_w_ =* (*m* − 1)*τ*. The method of C-C [26] is chosen to determine *m* and *τ* in this section. 

The steps of TRSWA-BP and PSR for forecasting are described as follows.

1. Select the same five sequences as step 1 in Section 2.2.
2. Determine the *m* and *τ* of the above sequences through the C-C method.
3. The sequences of *v*(*t_i_*), *d*(*t_i_*), *p*(*t_i_*), *tep*(*t_i_*), and *v_NWP_*(*t_i_*) are reconstructed, respectively, based on Equation (5), into the following sequences:
  $$\left\{v(t_{i}),v(t_{i}+\tau_{1}),\ldots ,v(t_{i}+(m_{1}-1)\tau_{1})\right\}$$
  $$\left\{d(t_{i}),d(t_{i}+\tau_{2}),\ldots ,d(t_{i}+(m_{2}-1)\tau_{2})\right\}$$
  $$\left\{p(t_{i}),p(t_{i}+\tau_{3}),\ldots ,p(t_{i}+(m_{3}-1)\tau_{3})\right\}$$
  $$\left\{tep(t_{i}),tep(t_{i}+\tau_{4}),\ldots ,tep(t_{i}+(m_{4}-1)\tau_{4})\right\}$$
  $$\left\{v_{NWP}(t_{i}),v_{NWP}(t_{i}+\tau_{5}),\ldots ,v_{NWP}(t_{i}+(m_{5}-1)\tau_{5})\right\}$$

Take the five sequences as inputs of TRSWA-BP for forecasting, and obtain the predicted wind power at the *k* moment.

4. Set *k* = *k* + 1. Add the new actual value into the time series and replace the earliest one to form scrolling sequences over time. Repeat steps 2 to 5 until the termination condition is satisfied.

### 2.4. TRSWA-BP Combined with EMD-Based PSR

**Figure 1:**
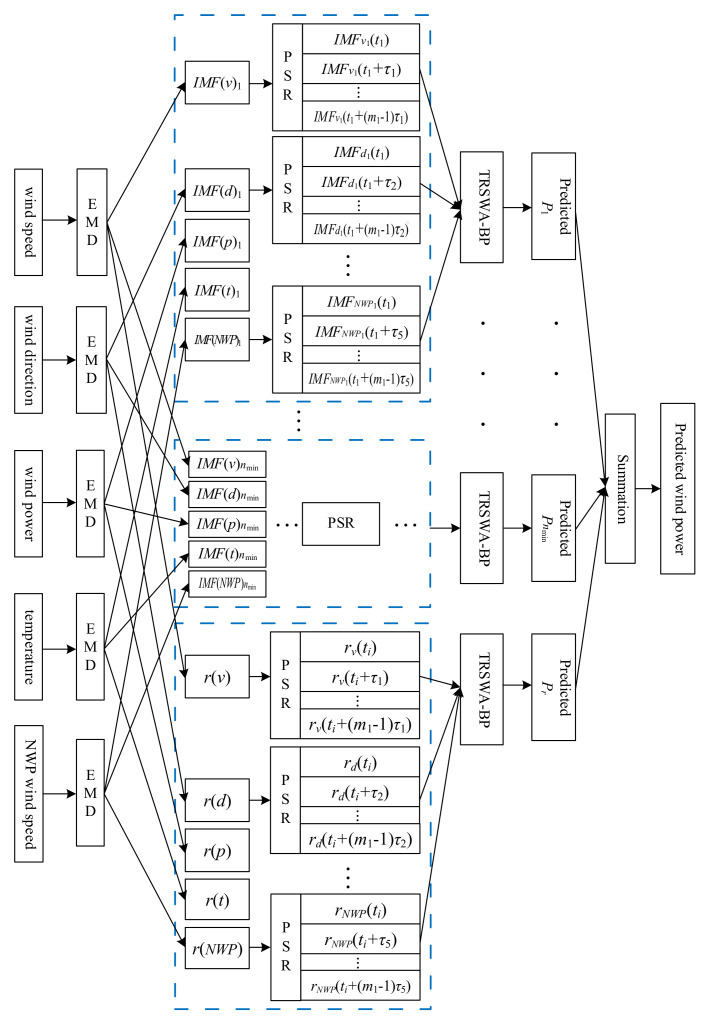
Five-input structure of the TRSWA-BP and empirical mode decomposition (EMD)-based phase space reconstruction (PSR).

As stated above, it may improve the accuracy theoretically when adding EMD or PSR processes into the prediction of TRSWA-BP. However, as for the IMF and residual component decomposed by EMD, the change of a variable in the dynamic system is related to its interaction with other variables. Consequently, it is possible to perform PSR after EMD. Figure 1 is the structure of the TRSWA-BP and EMD-based PSR that combines TRSWA-BP with these two methods, when taking the five sequences as inputs.

## 3. Entropy Evaluation for Error Sequence on Non-Gaussian Property

### 3.1. Non-Gaussian Property of Wind Power Error Sequence

The signal whose probability density function (PDF) is a non-normal distribution is collectively called the non-Gaussian signal. The non-Gaussian signal is usually described by skewness (*S*) and kurtosis (*K*) in engineering. Skewness measures the degree of random signals deviating from the symmetrical distribution signals. Signals with non-zero skewness must follow an asymmetric distribution. Kurtosis indicates the approximate state when the statistical frequency approaches the center of the distribution. Generally, the skewness and kurtosis of Gaussian random processes are zero, but at least one of them is not zero for non-Gaussian random process.

**Figure 2:**
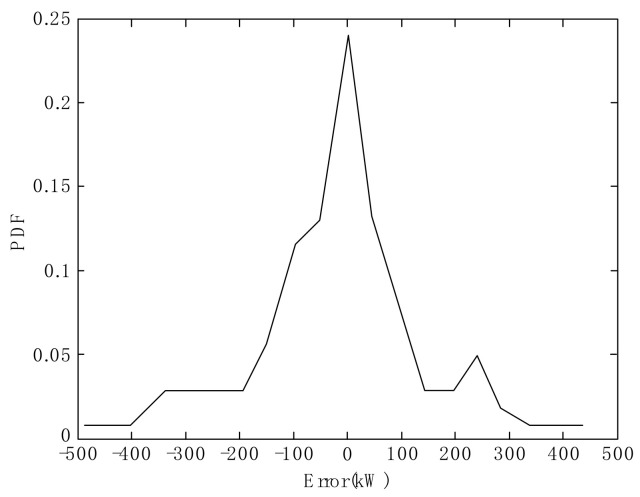
Probability density function (PDF) with non-Gaussian property of the forecasted power error.

In this section, suppose that *y_r_* is the measured output of wind power, while *y_f_* is its forecasted value, and assume that the estimated error *e* = *y_r_* − *y_f_* is a random variable. Based on forecasting by the model of TRSWA-BP, we give the PDF trend of estimated error sequence in Figure 2. We calculate the skewness and kurtosis of the error to give *S* = −0.5011 and *K* = 1.6722, hence both are non-zero. It is therefore demonstrated that the error sequence of wind power has non-Gaussian properties.

Generally, the PDF of the forecasted error is directly related to the prediction model. The NMAE and NRMSE are recognized as the evaluation criteria suitable for Gaussian distributions [27], which cannot fully reflect the randomness characteristic in wind power forecasting systems. The work we will concern ourselves with next is to develop the effective criteria for evaluating the influence of the non-Gaussian disturbances in wind power.

### 3.2. Evaluation Criterion Based on Normalized Renyi’s Quadratic Entropy

Entropy is a natural extension beyond mean square error because it is a function of PDF, which can provide a much more comprehensive description of the system as a measure of uncertainty, when compared with the variance. One of the most important problems for minimum entropy expression is the formulation of the system’s PDF. The entropy in the non-Gaussian case includes all higher-order information of random variables. Fortunately, Renyi’s quadratic entropy (RQE) is an effective method for the expression of non-Gaussian systems. Upon conclusion of Section 3.1, the error sequence of wind power presents the dynamic transitional changes at discrete data points {*e_i_*, *i* = 1, 2, …, *N*}. Consequently, a discrete form of *f_d_*(*e*) is adopted as Equation (6) [28], and its discretized Renyi’s quadratic entropy is derived as Equation (7).

(6) $$f_{d}(e)=\frac{1}{N}\sum_{i=1}^{N}G_{\Sigma }(e-e_{i})$$

(7) $$\begin{array}{ll} H_{d}(e) & =-\log(V_{d}(e)),\\ V_{d}(e) & =\int f_{{}_{d}}^{2}(e)de\\ & ={\int \left(\frac{1}{N}\sum_{i=1}^{N}G_{\Sigma }(e-e_{i})\right)}^{2}de\\ & =\frac{1}{N^{2}}\int \left(\sum_{i=1}^{N}\sum_{j=1}^{N}G_{\Sigma }(e-e_{i})G_{\Sigma }(e-e_{j})\right)de\\ & =\frac{1}{N^{2}}\sum_{i}^{N}\sum_{j}^{N}\int G_{\Sigma }(e-e_{i})G_{\Sigma }(e-e_{j})de \end{array}$$

where *G_Σ_*(·) is defined as Equation (8).

(8) GΣ(e−ei)=(2π)−n2(det∑)−12×exp{−12(e−ei)T∑−1(e−ei)}

**Figure 3:**
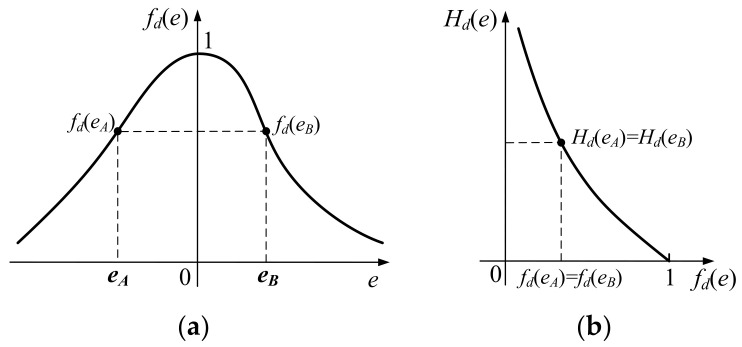
PDF calculations of the same *H_d_*(*e*) based on different errors *e_A_* and *e_B_* in a non-Gaussian distribution. (**a**) A PDF distribution obtained by the discretized Renyi’s quadratic entropy (RQE); (**b**) *H_d_*(*e*) is a monotone decreasing of *f_d_*(*e*).

Renyi’s quadratic entropy *H_d_*(*e*) is a monotone decreasing function of *f_d_*(*e*) [29], and the smaller the error *e* is, the larger the *f_d_*(*e*) should be. Nevertheless, for a PDF obtained by the discretized RQE, Equation (7) cannot fully reflect the error *e* because it contains two different values with one indicator. Figure 3 gives an example of this. If there are two different errors *e_A_* and *e_B_*, located in the positive and negative axes of *e* separately, with the same *f_d_*(*e*) in Figure 3a, we can only obtain the identical indicator *H_d_*(*e_A_*) = *H_d_*(*e_B_*) from Figure 3b. This means that the RQE is not a one-by-one correspondence between the error and its indicator, which will reduce the credibility of the RQE as an effective criterion, as it cannot accurately reflect the true situation of the dynamic error.

Accordingly, a new error evaluation criterion is built here as Equation (9), which is denoted as normalized Renyi’s quadratic entropy (NRQE) in the paper.

(9) $$H_{\mathrm{NRQE}}(e)=-\log\frac{1}{N}\sum_{i=1}^{N}e_{i}f_{d}^{2}(e)$$

where the dynamic error *e_i_* (*i* = 1, 2, …, *N*) is added to RQE to avoid confusing the indicators, *N* is the number of samples, and 1/*N* is used to balance the normalization of *H*_NRQE_(*e*).

**Figure 4:**
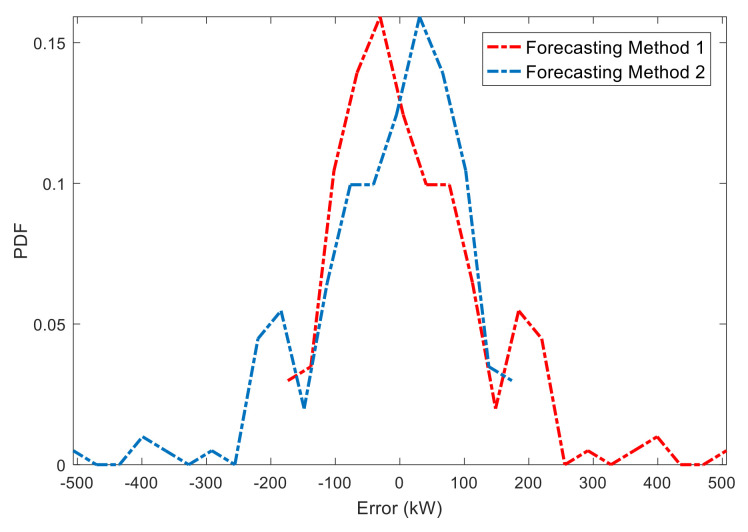
PDFs of the same positive and negative errors.

The following example will especially illustrate the superiority and applicability of the NRQE. Suppose that there are two prediction systems (forecasting methods 1 and 2 in Figure 4) that have the same absolute value of the errors, but one is positive while the other is negative. Their PDFs are obtained by fitting the discrete errors, as shown in Figure 4.

By taking the PDFs of the two systems into Equation (9), the calculation of their NRQEs as the evaluation criterion are −20.4 and 20.4, respectively, which can effectively distinguish their positive and negative deviation. However, the NMAEs and NRMSEs are both 8.7% and 11.5% in the same predictions, respectively, which cannot show the accurate deviations. In summary, the proposed NRQE can objectively reflect the dynamic errors that appear in uncertain predictions, and it will be introduced into a real evaluation system of the forecasted wind power in a wind farm.

## 4. Simulation and Analysis

### 4.1. Prediction and Evaluation Based on the TRSWA-BP

Data sequences from a wind farm in January 2015 are selected, in which the length *N* of training samples is 288, and that of forecasting is 150. The TRSWA-BP under 1, 4, 6, and 24 h time-scale forecasting is compared with the continuous method, ARMA, support vector machine (SVM), and BP. In the predictions, the input data is used by the continuous method and ARMA is only the wind power, and that concerned by SVM, BP, and TRSWA-BP are five sequences as mentioned in Section 2.2. The optimal parameters of TRSWA-BP are tested as follows: 5 inputs; 1 hidden layer with 13 nodes; 1 output; learning rate *η* = 0.2; and inertia coefficient *α* = 0.05. Meanwhile, evaluations used by the normalized mean absolute error (NMAE), normalized root mean square error (NRMSE), and NRQE are adopted to value the precision of the predictions, which can be formulated as Equations (10) and (11).

(10) $$NMAE=\frac{1}{\bar{P}}\frac{1}{N}\sum_{i=1}^{N}\left|P-P_{i}\right|$$

(11) $$NRMSE=\frac{1}{\bar{P}}\sqrt{\frac{1}{N}\sum_{i=1}^{N}{\left(P-P_{i}\right)}^{2}}$$

where *P*, *P_i_* represent the actual and predicted power values, while the overbar indicates the mean over the sampling points.

Table 1 gives comparisons by the result of average running 20 times in order to avoid random errors.

As shown in Table 1, the NMAEs, NRMSEs, and NRQEs of the SVM, BP, and TRSWA-BP are significantly lower than those of the continuous method and ARMA. Upon evaluation of the SVM, BP, and TRSWA-BP, the precisions of NMAE and NRMSE are very close, hence it is difficult to determine which is superior. For instance, when the time scale is up to 24 h, the NMAEs of SVM, BP, and the TRSWA-BP are 14.872%, 11.324%, and 10.175%, respectively. However, their NRQEs are 88.303, 82.335, and 64.807. Obviously, the TRSWA-BP is a good model in desired horizons based on the evaluation of the NRQE. 

**Figure 5:**
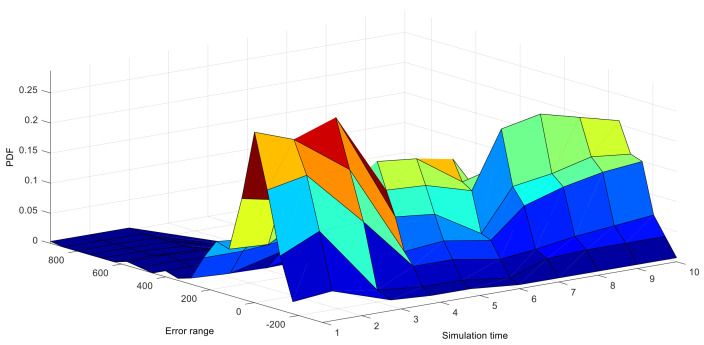
PDFs of the 10 h predicted errors, based on the model of TRSWA-BP.

We further verify the necessity of NRQE in 1–10 h upon 4-h-ahead predictions, once an hour. The PDFs of predicted errors, based on the TRSWA-BP, are dynamic, transitionally change along the time zone, and their distributions are non-Gaussian, as shown in Figure 5. It clearly expresses that the above criteria of NMAE and NRMSE, calculated by the static errors within a limited timescale, are not appropriate for the dynamic error functions. However, NRQE is much more suitable for its evaluation of stochastic wind power.

**Figure 6:**
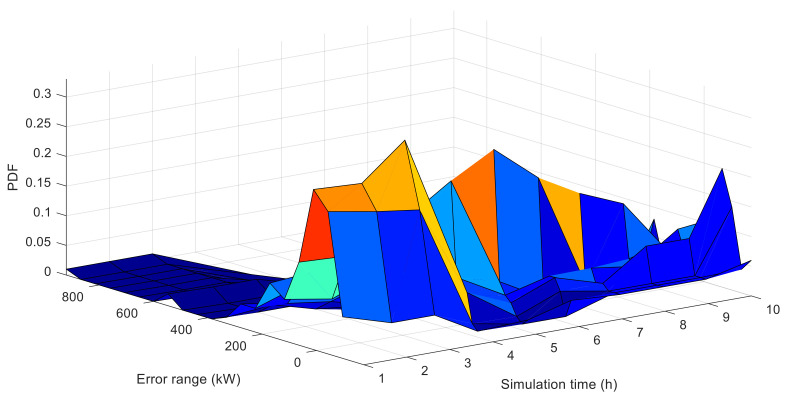
PDFs of the 10 h predicted errors, based on the model of BP.

**Figure 7:**
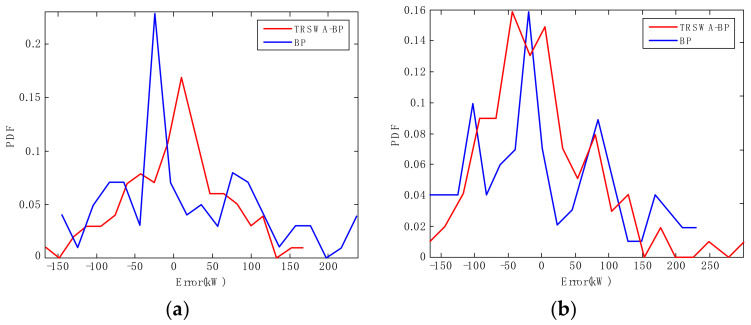
PDFs of the particular instant errors, based on two prediction methods. (**a**) At the 6 h instant; (**b**) at the 8 h instant.

A series of similar PDF curves, based on the BP model, are shown in Figure 6, which is under the same test conditions. It can be seen that the shapes of the two PDFs exhibit different characteristics at some particular instants. For example, at the 6 h instant, Figure 7a demonstrates that the PDF of the BP error becomes narrower and sharper, while the TRSWA-BP becomes fatter and smoother. At the 8 h instant in Figure 7b, the opposite phenomenon occurs. Therefore, a combined prediction of wind power can be suggested, based on the uncertainty evaluation of the NRQE.

### 4.2. Prediction Precision Based on the TRSWA-BP Combined with EMD & PSR

The models of TRSWA-BP combined with EMD, PSR, and EMD-based PSR are tested for the predictions, of which the time-scales are 1 h, 4 h, 6 h, and 24 h. Only the TRSWA-BP and EMD employs wind power as one input, while other models employ five inputs. In addition, comparisons of the predicted precisions among different models are shown in Table 2.

As shown in Table 1 and Table 2, the NMAE, NRMSE, and NRQE of TRSWA-BP and EMD have better performance than those of TRSWA-BP and TRSWA-BP and EMD (one input). Specifically, when the time scale is one h, the difference of NRQE is 11.352 between TRSWA-BP and EMD (2.439), and TRSWA-BP and EMD (one input) (13.791), and this difference increases as the time scale grows. Therefore, it is necessary to use more data sequences as EMD inputs.

When we make a choice for five-input sequences, the TRSWA-BP combined with EMD or PSR in Table 2 can significantly improve accuracy, when compared with the TRSWA-BP in Table 1, which is based on three evaluation criteria. However, the precision is not easily distinguishable from the rate of NMAEs and NRMSEs between the models compared. The evaluation criteria of the NRQEs that reflect the true situation of dynamic errors is more feasible upon the models.

### 4.3. Training Times

**Figure 8:**
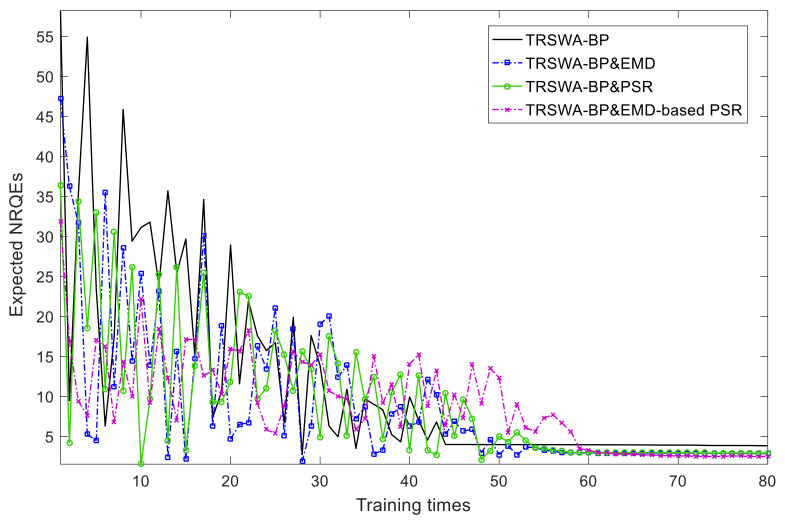
Expected NRQEs of the proposed networks.

For empirically comparing the convergence rate, five-input sequences are used to train the four proposed networks, based on the NRQEs. The results are plotted in Figure 8.

From Figure 8, it can be seen that as the expected NRQEs reduce, the training times of the four networks increase rapidly. When the training times vary from 40 to 70, the errors of the networks tend to gradually stabilize, in which TRSWA-BP has a minimum number of trainings at 23, and TRSWA-BP and EMD-based PSR has a minimum steady-state error, but experienced the longest trainings. 

**Figure 9:**
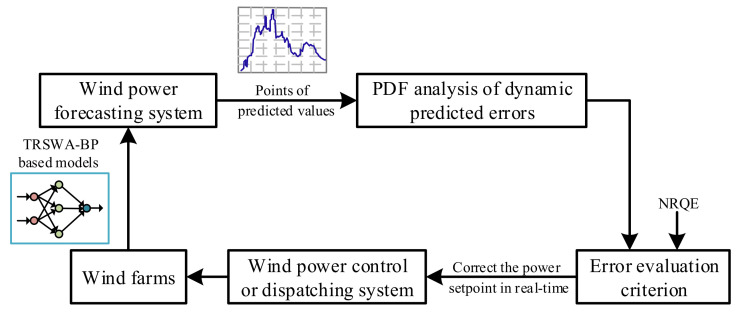
Wind power control or dispatching system with NRQE evaluation.

In summary, from these figures we can observe that: (1) The basic model of TRSWA-BP is a fast convergence algorithm in desired horizons; (2) The accuracy of the predictions combined with EMD, PSR, and EMD-based PSR are acceptable, and can effectively revise the error due to the fluctuation property of wind power; (3) The model of TRSWA-BP and EMD-based PSR gives the greatest accuracy with more training times; and (4) The NRQE illustrates its comprehensive evaluation of the transitionally changed errors that appear in uncertain predictions, which can be applied to the future minimum tracking error control for the closed-loop system, with a random disturbance that is shown in Figure 9.

## 5. Conclusions

A TRSWA-BP model is proposed in this paper, which has a competitive accuracy when compared with the continuous method, ARMA, SVM, and BP in short-term forecasting of wind power. Considering the strong intermittency and multifractal properties of wind power, TRSWA-BP combined with EMD and PSR is further established to weaken the influence of volatility. Although EMD and PSR are not the best choices when solving online modeling problems, the training times in expected errors are still in an acceptable frame.

Under detailed analysis of the non-Gaussian disturbances in stochastic wind power, a novel evaluation criterion of normalized Renyi’s quadratic entropy (NRQE) is proved to be effective in assessing the uncertain and dynamic predicted error. The NRQE can distinguish positive and negative deviations, and is much more favorable for combined forecasting. It is evidenced that the NRQE is a good candidate criterion on error evaluation, and ready for further minimum tracking for the use of stochastic error in wind power control.

Further research should focus on the following: (1) Experimental effectiveness is verified with more data and models from different wind farms; (2) concern and evaluate brief structures in models, and give them good practice to keep the code concise; and (3) based on the criterion of NRQE, a variety of forecasting methods is optimized to establish a decision support system.

## Figures and Tables

**Table 1:** Comparison of predicted precision based on different models.

Predictable Time Scales		1 h	4 h	6 h	24 h
Continuous method	NMAE	7.929%	15.384%	16.998%	20.579%
	NRMSE	9.784%	19.718%	24.136%	24.712%
	NRQE	17.967	99.572	108.77	110.963
ARMA	NMAE	7.117%	15.244%	18.729%	23.411%
	NRMSE	10.258%	20.012%	23.881%	27.160%
	NRQE	17.845	94.533	104.686	123.148
SVM	NMAE	6.090%	9.108%	11.153%	14.872%
	NRMSE	9.488%	10.359%	15.025%	18.431%
	NRQE	14.333	25.959	38.65	88.303
BP	NMAE	5.533%	7.515%	9.110%	11.324%
	NRMSE	9.817%	12.599%	14.890%	17.471%
	NRQE	7.545	25.997	46.323	82.335
TRSWA-BP	NMAE	5.218%	6.782%	8.080%	10.175%
	NRMSE	8.144%	10.779%	14.033%	17.029%
	NRQE	5.945	20.442	38.422	64.807

**Table 2:** Predictions based on three evaluation criteria.

Forecasting Time Scales		1 h	4 h	6 h	24 h
TRSWA-BP and EMD (one input)	NMAE	7.487%	9.891%	11.716%	13.245%
	NRMSE	8.363%	10.264%	13.464%	15.758%
	NRQE	13.791	40.347	54.763	91.077
TRSWA-BP and EMD	NMAE	6.122%	8.325%	9.898%	11.652%
	NRMSE	7.248%	10.029%	12.425%	14.780%
	NRQE	2.439	17.967	23.890	58.461
TRSWA-BP and PSR	NMAE	6.311%	7.359%	8.870%	10.543%
	NRMSE	7.524%	9.855%	12.220%	13.668%
	NRQE	5.144	18.855	23.592	56.922
TRSWA-BP and EMD-based PSR	NMAE	5.257%	6.818%	8.131%	9.755%
	NRMSE	7.245%	8.920%	10.488%	13.177%
	NRQE	1.522	6.378	22.667	56.480
